# Are Protein Cavities and Pockets Commonly Used by Redox Active Signalling Molecules?

**DOI:** 10.3390/plants12142594

**Published:** 2023-07-09

**Authors:** John T. Hancock

**Affiliations:** School of Applied Sciences, University of the West of England, Bristol BS16 1QY, UK; john.hancock@uwe.ac.uk; Tel.: +44-(0)1173282475

**Keywords:** argon, hydrogen peroxide, hydrogen sulfide, hydroxyl radicals, molecular hydrogen, nitric oxide, peroxynitrite, protein cavities, superoxide, xenon

## Abstract

It has been well known for a long time that inert gases, such as xenon (Xe), have significant biological effects. As these atoms are extremely unlikely to partake in direct chemical reactions with biomolecules such as proteins, lipids, and nucleic acids, there must be some other mode of action to account for the effects reported. It has been shown that the topology of proteins allows for cavities and hydrophobic pockets, and it is via an interaction with such protein structures that inert gases are thought to have their action. Recently, it has been mooted that the relatively inert gas molecular hydrogen (H_2_) may also have its effects via such a mechanism, influencing protein structures and actions. H_2_ is thought to also act via interaction with redox active compounds, particularly the hydroxyl radical (^·^OH) and peroxynitrite (ONOO^−^), but not nitric oxide (NO^·^), superoxide anions (O_2_^·−^) or hydrogen peroxide (H_2_O_2_). However, instead of having a direct interaction with H_2_, is there any evidence that these redox compounds can also interact with Xe pockets and cavities in proteins, either having an independent effect on proteins or interfering with the action of inert gases? This suggestion will be explored here.

## 1. Introduction

It is well recognised that under stress conditions, cells produce a range of small signalling molecules [1,2]. Many of these are relatively reactive and have redox activity. Compounds that accumulate in cells include reactive oxygen species (ROS [3]), such as hydroxyl radical (^·^OH), superoxide anions (O_2_^·−^), or hydrogen peroxide (H_2_O_2_), as well as reactive nitrogen species (RNS), such as nitric oxide (NO^·^ [4]) and peroxynitrite (ONOO^−^). These compounds are produced by dedicated enzymes. This includes NADPH oxide [5] for ROS (producing superoxide, which can cascade in reactions to generate H_2_O_2_, for example), xanthine oxidoreductase (producing H_2_O_2_ or NO depending on oxygen (O_2_) concentrations [6]), and peroxidases metabolising H_2_O_2_ [7]. RNS are produced by nitric oxide synthase (NOS [8]), of which there are three isoforms in humans, and nitrate reductase (NR), which is important for plant NO generation and metabolism [9]. Hydrogen sulfide (H_2_S) is also used in stress responses and is produced by a range of enzymes in mammals: cystathionine γ-lyase (CSE), cystathionine β-synthase (CBS), and 3-mercaptopyruvate sulfurtransferase (3-MST) [10].

Many of these signalling-active compounds are relatively toxic, so they have a detrimental effect on cells at high concentrations. For example, H_2_S has effects on mitochondrial function, which partly accounts for its toxicity [11]. However, at low concentrations, these molecules have significant signalling roles, which allow for stress tolerance, including during drought [12], pathogen challenge [13], salt stress [14], heavy metal stress [15], and extreme temperature tolerance [16].

The reactivity of ROS, RNS, and H_2_S partly accounts for the cellular responses seen. Many proteins contain one or more relatively reactive thiol groups as side chains of cysteine residues. Such -SH groups can be deprotonated to the thiolate. If two cysteines are in the correct three-dimensional orientation, they can react and combine to create a disulfide cystine. This can add to the structural rigidity of a protein. On the other hand, thiols can be modified by a reaction with a range of reactive signalling molecules. H_2_O_2_ can lead to the oxidation of thiol to create the sulphenic acid group [17], a modification that is reversible, so in a manner akin to phosphorylation, this oxidation can create a new protein topology and hence activity, which can be readily reversed. Higher H_2_O_2_ concentrations can lead to higher oxidation states of the thiol, i.e., suphinic acid and sulphonic acid, and this leads to irreversible modification. NO leads to the modification of thiols to produce the -SNO group (*S*-nitrosylation [18]), which can be reversed in a similar manner to phosphorylation. H_2_S leads to the persulfidation [19] of thiols to produce -SSH, which is reversible. Such thiol modifications of proteins can account for the range of effects seen on ROS/RNS/H_2_S accumulation. These reactive molecules can also react together and create new reactive signalling molecules. Superoxide and NO can react, for example, to create peroxynitrite, a signalling molecule in its own right [20]. NO and H_2_S will react to generate nitrosothiols [21], which can also act in a signalling role. Therefore, the reactivity of these molecules is important for their action in signalling. 

Molecular H_2_ is also known to have signalling effects and, therefore, needs to be considered alongside other small signalling molecules. H_2_ has been shown to have effects in a range of disease conditions in humans, such as diabetes [22], degenerative disease [23], and even COVID-19 [24]. H_2_ has been mooted as being reactive with hydroxyl radicals (^·^OH) and peroxynitrite [25]. It is thought to be unreactive with O_2_^·−^, H_2_O_2_, or NO. It seems unlikely that H_2_ has all its effects via interactions with ^·^OH or ONOO^−^, and therefore some other mechanisms of action are probably pertinent to consider. H_2_ may act via its redox action, for example, as argued previously [26]. 

It has been long known that inert gases such as xenon (Xe) [27] have biological effects. Inert gases such as Xe will not chemically react with biomolecules such as proteins; therefore, they must have a different mode of action. There has been a body of work on how Xe (and other inert gases) interact with polypeptides, and it is known that they may engage with cavities and pockets in proteins, as discussed in more depth below. The recent work by Turan et al. [28] on myoglobin (Mb) exemplifies this kind of work. Although such binding and interaction of inert gases has been studied in animal systems, such as the Mb work, there are examples where work has been carried out in plants. For example, Duff et al. [29] worked on plant copper amine oxidases, while Murray et al. [30] investigated the binding of Xe to photosystem II. Having said that, there is no reason why the binding of inert gases, and as suggested here, ROS/RNS, etc., could not be a common mechanism in any organism. Therefore, the discussion is not limited to plants, and it is hoped that this discussion will be of interest to a range of biochemists.

With the knowledge that Xe can interact with proteins, it has recently been suggested that H_2_ may mimic the action of other gases, i.e., it may interact with cavities and pockets in the structures of proteins [31]. Therefore, examining how inert gases act may provide an indication of how other molecules may have bioactivity. Following on from this, a question that could be posed here is: does this only apply to inert gases? Can other small signalling molecules, whether inert or not, have a similar mode of action? 

**Figure 1:**
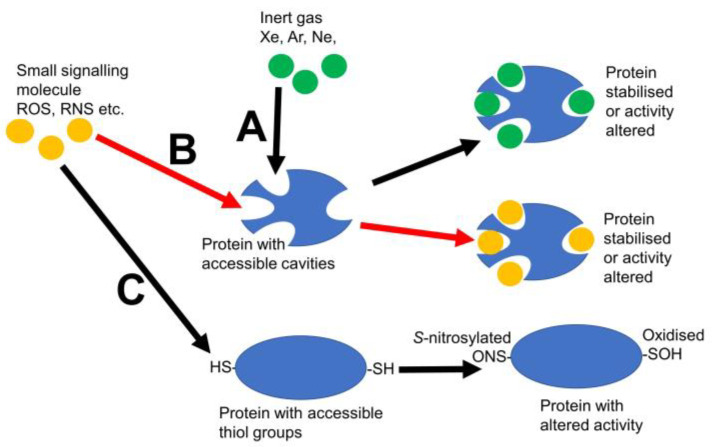
Possible modes of action of inert gases and other small signalling molecules. (**A**) Inert gases acting via their interaction with protein cavities. (**B**) Small signalling molecules such as ROS and RNS acting through their interaction with protein cavities. (**C**) Small signalling molecules such as ROS and RNS acting via their interaction with thiol groups on proteins. Black arrows indicate mechanisms that are well recognised. Red arrows indicate a mechanism mooted in this paper.

Here, it is suggested that small signalling molecules can act in a manner akin to Xe. The actions and ideas mooted here can be summed up in the scheme shown in Figure 1. The discussion below explores these ideas more deeply and looks at some of the evidence. If small signalling molecules such as ROS and RNS can work via this mechanism, such discussion will unlock a new way of looking at the mode of action for a range of hugely important signals in all organisms, including plants. 

## 2. Other Inert Gases

The classical inert gas that is known to have bioactivity is Xe. This has been known for a long time, at least since the 1940s [27]. Some of the actions of Xe include behaving as an anaesthetic and having cytoprotective effects [32]. There seems to be no doubt that Xe has physiological effects and acts on cellular function. However, if Xe is inert, how does it interact with biomolecules? 

Proteins are known to have complex, dynamic, and often quite loose structures, which include cavities and pockets in their topology. In these cavities and pockets, some small molecules may migrate. It is interesting to note that such cavities are often dubbed “Xenon binding pockets”, for example, in an article by Duff and colleagues [29]. However, this does not mean that these cavities are exclusively occupied by Xe or that the interaction of the small atom/molecule is a static event. Recent work by Turan et al. [28] shows that the Xe can migrate through channels in the protein, in this case Mb, and therefore there is a dynamic interaction taking place. Indeed, this work shows that Mb is in fact an allosteric protein. 

The globin family has been used as a model system for studying Xe binding. This interaction has been known for a long time. For example, Schoenborn et al. [33] explored Xe binding to sperm whale Mb in the 1960s. Researchers continued to understand the interactions more, for example, the work in the 1980s by Herman and Shankar [34] and Tilton et al. [35], and has continued to today, as exemplified by the work by Turan and colleagues [28]. Research with haemoglobin has a similar history, with reports spanning back to at least the 1960s [36], work taking place in the 1980s [37], and more recent work being reported [38].

However, Xe interacts with a range of proteins. Prangé et al. [39] list a range of proteins that were studied for the binding of Xe and Krypton (Kr). These include elastase, subtilisin, cutinase, collagenase, lysozyme, the lipoamide dehydrogenase domain from the outer membrane protein P64k, urate-oxidase, and the human nuclear retinoid-X receptor RXR-α. Rubin et al. studied maltose binding protein [40], while others have focused on copper amine oxidases [29,41] and urate oxidase [42]. It has also been found that in humans, Xe leads to an increase in erythropoietin by triggering an increased production of hypoxia-inducible factor 1α (HIF-1α) [43]. Clearly, there is a range of proteins that are able to bind inert gases, with the potential for changes in their structures and therefore functions. In this vein, Winkler et al. have reported in silico screening to identify xenon protein targets [44]. 

However, Xe is not the only inert gas that is known to have an influence on a cell’s activity. Kr was mentioned above [39]. As with other gases, this work spans back to at least the 1960s, when the solubility of Kr in biological materials was reported [45], whilst more recent work has shown that Kr nanobubbles inhibit the activity of pepsin [46]. Argon (Ar) is known to have neuroprotective [47] and organoprotective effects [48]. For example, it was shown to be neuroprotective against cerebral ischemia and brain injury in in vitro models [49]. Ar appears to have neuroprotection by inhibiting Toll-like receptors, and it also has anti-apoptotic action [50]. Some of Ar’s effects may also be due to altering kinase signalling in neurons and glial cells [51]. Ar is also known to have narcotic effects. A review of argon’s biological effects was published by Ye et al. [52], but what seems certain is that Ar has effects on proteins despite being inert.

Other inert gases worth considering here are Neon (Ne) and helium (He). Both have reported effects [53]. However, just because one gas has an effect does not mean they all do. Interestingly, in *Neurospora crassa*, it was reported that the growth rate in the presence of inert gases was correlated to the molecular weight of those gases. There was also a formula provided: R = 3.88 − 0.1785 (MW)^½^, where R is the growth rate (in millimetres per hour at 30 °C) [54]. In a neuronal injury model, Xe was found to be protective, Ar and Kr had no effect, and He was the detrimental effect [55], a trend also reported by Rivzi et al. [56]. Therefore, what is found with one gas cannot be automatically translated to the other gases. This needs to be borne in mind if we are going to extend this idea of small molecules binding to proteins to a range of other compounds, such as ROS, RNS, or H_2_S.

As already mentioned, H_2_ could be thrown into this mix [31]. There is no evidence that H_2_ has an influence on the activity of proteins using such a mechanism. There are issues to be considered here. H_2_ is extremely small, and perhaps it could be argued that it is too small to have such an effect. On the other hand, there is no reason to suspect that single H_2_ molecules act alone, and until a thorough investigation of this possible mechanism of H_2_ is carried out, there is no reason to rule this out, or indeed in, as a mode of action of H_2_.

## 3. Other Small Signalling Molecules That May Need to Be Considered When Discussing Protein Cavities

It is known that inert gases can interact with proteins via cavities and pockets. Here it is suggested that other small molecules, especially ROS, RNS, and sulphur-based molecules (so-called reactive sulfur species (RSS)), should be considered. Classically, these molecules are grouped together, but in reality, they are a wide range of compounds. Xe has a Van der Waals radius of 216 pm, so anything smaller than this should be able to partake in the protein interactions suggested here. Any molecule that is substantially larger may be excluded from cavities and pockets, but there is also the inherent reactivity of the molecule to consider. 

The term ROS traditionally embraces those molecules based on oxygen [57]. Therefore, O_2_^·−^ is to be considered here. This molecule is relatively unstable and is likely to react with other molecules. However, it can also be protonated and move through membranes. It is small and should be considered here. O_2_^·^ can readily dismute to H_2_O_2_, a molecule known to have numerous signalling roles in a range of organisms [58], and therefore should be included here. On the other hand, although small, ^·^OH is very reactive and unlikely to partake in sitting in protein pockets without having an imminent or previous chemical reaction. Other ROS that would be worth considering include other peroxides, singlet oxygen (^1^O_2_) [59], and perhaps alpha oxygen (α-O), both of which would be small enough to enter protein pockets. 

RNS are also a wide-ranging group of compounds, and like ROS, they are known to have signalling functions in cells [60]. Although NO is often the focus as it is such an important signalling molecule [61]. However, as mentioned, superoxide and nitric oxide can react together to produce ONOO^−^, a relatively reactive but also relatively small molecule. It should probably be considered a compound worth more investigation here. However, there are also several other RNS worth considering, including nitrosyl anions, nitrosyl cations, nitrogen dioxide, dinitrogen oxide, and nitronium ions.

Small signalling molecules based on sulphur are also a diverse range of molecules, some of which are likely to have signalling roles in cells [62,63]. Most of the signalling roles of this group of compounds tend to focus on H_2_S [64]. However, several other compounds ought to be considered for discussion here. These include the sulfhydryl (HS^·^) or thiyl radicals (RS^·^), but these may be too reactive to interact with protein cavities as suggested here. Sulfur compounds can also produce disulphides. Hydrogen disulfide (HSSH) may be worth considering, but those with large R groups (RSSR) are less likely to interact with cavities simply because of steric hindrance. Glutathione (GSH or GSSG) is also unlikely to be important here either, for the same reason. Other RSS that ought to be contemplated are thiosulfate, polythionates, and even perhaps elemental sulfur [65].

What is clear, however, is that there is range of compounds that are small and have biological effects, and as such, it would be worth considering many of these as acting in the same manner as Xe. 

## 4. Do Other Small Signalling Molecules Use Xe Pockets?

It is clear that Xe is not the only gas that takes advantage of the cavities and channels in proteins. It is likely that other inert gases, e.g., Ar, Kr, and He, may have the same mode of action. However, the question being asked here is: can this mechanism of direct interaction with protein structures be extended to the action of small molecules that are known to have profound effects on cell signalling? Some of these candidate molecules are themselves gases, such as NO and H_2_S, so perhaps this is not such a stretch to consider. 

There are certainly some examples in the literature that would support small molecules interacting with Xe-binding pockets. For example, in haemoglobin, oxygen has been shown to migrate through Xe docking sites while the protein is in the R-state [38]. Previously, using mutations in the protein, Scott and Gibson [66] had looked at the effects of Xe on O_2_ binding to Mb. Furthermore, the migration and escape of both O_2_ and carbon monoxide (CO) from Mb seem to take advantage of Xe-binding regions and are influenced by the presence of Xe [67]. 

Nitrous oxide has effects on proteins and, in many cases Xe is used as a model or a method of investigation [42,68,69]. Using urate oxidase as a model, Marassio et al. [42] report that “Xe and N_2_O bind to, compete for, and expand the volume of a hydrophobic cavity” in the protein, and so this leads to the inhibition of activity. Later, the same group [68] argues that although both gases bind to proteins, the mechanisms are not the same. With a focus on P450 monooxygenase via the work of LeBella et al. [69], it was shown that both Xe and nitrous oxide occupy a haem-pocket in the enzyme and hence lead to inhibition. Therefore, here we have a gas that does not sit in the noble gas group but does have biological activity and seems to have a mechanism akin to that of the noble gases. 

If such a mechanism can mediate the effects of nitrous oxide, is there scope to consider such a mechanism for other small signalling molecules?

In what are described as “gas pockets”, Winter et al. explore how nitric oxide and other gases have their binding facilitated by the presence of hydrophobic cavity regions [70]. It has been suggested that NO can bind momentarily to alternate cavities in Mb. These cavities are some distance from the haem binding site and therefore not near the O_2_ binding site [66,71,72]. Brunori [71] goes on to say that the sites where NO bind are those that have been identified in Mb as binding Xe [73]. In haemoglobin, it appears that NO has movement through tunnels in the protein structures. The paper discusses the presence of short and long tunnels and emphasises that hydrophobic residues at the entrances to such tunnels are important: Phe for the long tunnel and Ile for the short tunnel. The authors also point out that NO can “diffuse from Xe cavity to Xe cavity” [74]. NO has been shown to bind to the haem-pocket in horse radish peroxidase [75]. In a bacterial system, it is thought that there is an interaction between nitrite reductase (NiR) and NO reductase (NOR), but of pertinence to the argument here, NO generated by the first enzyme (NiR) migrates to NOR, with that movement being facilitated by the NO translocating through a cavity in NiR and then a hydrophobic channel in NOR [76].

For other gases, such as carbon monoxide (CO), a similar situation has been reported. Chu et al. [77] asked if the binding of gases such as NO, O_2_, CO, or H_2_ is a random event on proteins but concluded that the migration of such ligands involves a limited number of pathways and is facilitated by the presence of specific docking sites on the protein [77]. Elber and Karplus [78] suggested that in Mb, CO used Xe binding regions, a notion that more recently has been revisited and reported on [79]. In sperm whale Mb, Bossa et al. [80] also suggested that CO takes advantage of Xe pockets for binding and migration through the proteins, along with what they describe as “additional packing defects”. Others too have added to the weight of evidence that CO migrates through proteins such as Mb, facilitated by Xe-binding regions [81]. Using nitrogenase, it was suggested that CO might migrate through the protein through a gas channel which is composed of a series of cavities [82]. Using CO as a probe in hydrogenases (*Dd*HydAB from *Desulfovibrio desulfuricans*; *Ca*HydA from *Clostridium acetobutylicum*; *Cr*HydA1 from *Chlamydomonas reinhardtii*), it was shown that inhibition was dependent on the redox state of the H cluster but also the migration of the gas through the protein [83]. 

With the view to understanding how proteins may be useful for carbon capture—with the background of climate change—Cundari et al. [84] suggested that CO_2_ binding to proteins is facilitated by acid/base interactions and that β-sheet structures are better than α-helices.

What of ROS, which are thought to have many of their effects via thiol modification? Using 4-hydroxybenzoate hydroxylase (PHBH) and phenol hydroxylase (PHHY), Hiromoto et al. [85] suggested that their data implied that hydrophobic pockets served as binding sites for H_2_O_2_. In cholesterol oxidase, it was found that both O_2_ and H_2_O_2_ are able to interact with a hydrophobic tunnel in the protein [86]. Zhao et al. [87] have taken this idea further and engineered tunnels in cytochrome P450 monooxygenases that are able to accommodate H_2_O_2_. Superoxide anion migration was seen, a process that was reliant on the presence of a tyrosine residue [88]. Therefore, examples of how ROS can, and need to, interact with proteins via direct physical mechanisms have been reported. 

## 5. Conclusions and Future

Many gases, such as Xe, have anaesthetic effects, and this has been known for a long time. As pointed out by Eckenhoff [89], Claude Bernard suggested in 1875 that such effects involved proteins. Eckenhoff also cites papers pointing out that the presence of hydrophobic domains in proteins where inert molecules could interact has also been known for a long time [90,91]. Such work is still continuing, as exemplified by the work of Colloc’h et al. [92] and Turan et al. [28]. 

There seems little doubt that inert gases such as Xe have an influence of protein activity [39], and therefore the activity of the cell, via the direct interaction of the gas molecules with proteins by taking advantage of the pockets and cavities that exist in protein structures. Other inert gases have similar biological effects and actions, including Ar, He, and Ne, while it has been suggested H_2_ also acts in this way [31]. Certainly, for H_2_, much more work focused on this potential mechanism needs to be carried out, either to confirm that this is one of the modes of action of H_2_ or to rule it out. Recent work has concentrated on the interaction of H_2_ with haem and the subsequent effects mediated by the removal of hydroxyl radicals [93,94,95]. However, such mechanisms probably do not account for all the actions of H_2_. Several modes of action probably need to be considered to obtain a full understanding of what H_2_ is doing in cells [31], including H_2_ interactions with protein cavities.

Other gases, such as nitrous oxide, appear to use Xe binding sites for their action [42,68,69]. However, what about other small signalling molecules, which fall under the umbrella terms ROS, RSS, and RNS? Although many such small reactive molecules, such as H_2_O_2_ and NO, have other mechanisms by which they interact with proteins, such as oxidation [17] and *S*-nitrosylation [18], respectively, little is known about how such molecules may interact with proteins by exploiting physical interactions, such as hydrophobic regions, cavities, and pockets, in the manner in which Xe acts. However, there is some evidence for NO, H_2_O_2_, and O_2_^·−^ interacting with proteins by exploiting Xe-pockets and similar structures [85,86,87,88]. 

A series of questions can be raised here. Should such interactions be more thoroughly investigated? Is there competition between small signalling molecules at such interaction sites in proteins? After all, many of these molecules will be present or even accumulating—such as during stress responses—together in cells. Is the binding of one signalling molecule more likely than others, i.e., is there a hierarchy of binding? This seems to be the case for the noble gases [54]. Can all such molecules partake in this sort of protein interaction? After all, perhaps H_2_ is too small. Or do these molecules manage to pack into these cavities and tunnels in some way, perhaps preventing others from interacting?

Here, there is an attempt to bring together some of the relevant literature about small molecules, some of which are inert and/or gases, interacting with proteins using polypeptide cavities and tunnels. It is hoped that this will inspire researchers to look outside of the normal paradigms of how ROS, RNS, and H_2_S may interact with proteins. The future may show that this is a fruitless exploit, but with inert gases such as Xe having profound effects on protein activity, it is suggested here that such protein biochemistry at least be considered. 

## Data Availability

No new data were generated for this article.
